# Familial hypercholesterolemia mutations in Petrozavodsk: no similarity to St. Petersburg mutation spectrum

**DOI:** 10.1186/1471-2350-14-128

**Published:** 2013-12-27

**Authors:** Tatiana Yu Komarova, Victoria A Korneva, Tatiana Yu Kuznetsova, Alexandra S Golovina, Vadim B Vasilyev, Michail Yu Mandelshtam

**Affiliations:** 1 Department of Molecular Genetics, Institute for Experimental Medicine, NW Branch of Russian Academy of Medical Sciences, Pavlov Street, 12, St.Petersburg 197376, Russia; 2 Department of Faculty Therapy, Infectious diseases and Epidemiology, Petrozavodsk State University, Lenin Street, 33, Petrozavodsk, Republic of Karelia 185910, Russia; 3 Department of Biochemistry, St.Petersburg State University, Universitetskaya nab., 7/9, St.Petersburg 199034, Russia

## Abstract

**Background:**

Familial hypercholesterolemia (FH) is a human monogenic disease induced by a variety of mutations with striking genetic diversity. Despite this variability recurrent mutations occur in each population studied, which allows both elucidating prevalent mutations and developing DNA diagnostic tools for the disease. Recent research of FH in St. Petersburg, Moscow and Novosibirsk (major cities in Russia) demonstrates that each megapolis has its own FH mutation spectrum sharing only small part of mutations with other populations in Russia and Europe. In order to optimize molecular-genetic diagnostic protocols for FH in Russia we studied mutation spectrum in other regions including Petrozavodsk, a smaller town in relatively close proximity to St. Petersburg.

**Methods:**

The principal method was automated detection of single-strand conformation polymorphism followed by direct PCR amplified DNA sequencing.

**Results:**

Twelve different mutations of the low density lipoprotein (LDL) receptor gene were detected in the Petrozavodsk sample (80 patients). Out of these twelve mutations, seven have never been described before (c.192_201delinsGGACTTCA, c. 195_196insT, c. 618 T > G, c. 1340C > G, c. 1686_1693delinsT, c. 1936C > A, c. 2191delG). Other five mutations (c. 58G > A, c. 925_931del, c. 1194C > T, c. 1532 T > C, c. 1920C > T) were previously characterized elsewhere. All new mutations are considered to be a probable cause of the FH in their carriers. Direct evidence of the neutral character of c.58G > A or *p. (Gly20Arg)* is provided for the first time. Each pathogenic mutation was a trait of its own unique pedigree and so far has not been found in other patients.

**Conclusions:**

Strikingly, out of twelve mutations characterized in the Petrozavodsk sample only one mutation, c. 925_931del, has previously been found in patients from St. Petersburg and Finland (most closely located studied populations), suggesting some common roots in origin of these populations in the past or limited gene exchange between them nowadays. No recurrent mutations were detected.

## Background

Familial hypercholesterolemia is a dominant inborn error of metabolism due to LDL lowered catabolism *via* specific LDL receptor. The disease is quite common (1:500) and can be diagnosed by detection of mutations in LDL receptor gene [1]. More than a thousand different mutations have been characterized in Europe [2-6], their spectra demonstrating ethnic specificity as well as importance of the founder effect. In Russia only patients from three cities: St. Petersburg [7], Moscow [8] and Novosibirsk [9], were studied showing high heterogeneity of the disease within the country. In order to search for ethnic mutations and to improve the DNA diagnostics of FH we carried out the mutation spectra characterization in the city of Petrozavodsk, Republic of Karelia, Russia.

## Methods

We collected DNA samples from 80 hypercholesterolemic patients from Petrozavodsk city demonstrating at least two of the following parameters: (1) positive family history of hypercholesterolemia and coronary heart disease; (2) high serum cholesterol, usually higher than 8 mM; (3) the presence of stigmata as corneal arcus, tendon xanthomata or xanthelasma. All patients signed an informed consent for the study. Research was performed in compliance with the Helsinki Declaration and approved both by Petrozavodsk State University and Institute of Experimental Medicine ethics committees. DNA was isolated from peripheral blood mononuclear cells nuclei using standard method with phenol-chloroform extractions [7,10]. All exons and promoter of the LDL receptor gene were amplified in separate PCR with the help of Cy-5 labelled oligonucleotide primers as described [7] and subjected to SSCP analysis on ALFExpress DNA automated sequencer (Life Sciences). Gels were interpreted with the help of ALFWin DNA Fragment Analyzer program. Samples demonstrating shifted mobilities in SSCP analysis were gel-purified and sequenced by Sanger method on contract terms at Eurogen Co. (Moscow).

## Results

Total of 18 sequence variations were found in the Petrozavodsk FH sample (Table 1). Lipid data of the patients with specific LDL receptor mutations are listed in Additional file 1. *APOB* R3500Q mutation common for FH in Europe was not found in FH patients from Karelia in the studied sample. For majority of variations in nucleotide sequence detected, rapid detection methods by RFLP analysis were developed (Table 1). Presence of the specific mutation was confirmed by restriction analysis using enzymes commercially available from Sibenzyme (Novosibirsk, Russia). The isoschizomers used and their prototypes are listed in Additional file 2. Effect of mutations on protein function was predicted using various programs *in silico* (Additional file 3) and by segregation analysis.

**Table 1:** List of mutations and polymorphisms in the LDL receptor gene in Petrozavodsk FH sample and methods of their detection

**Mutation, systematic name [name according to Yamamoto nomenclature] **[11]	**Nucleotide change**	**Exon/intron**	**Rapid test method**	**Occurrence in other populations **[3]**,**[5]	**Number of families (patients) with the mutation or rare allele frequency**
**Missense-mutations**					
*p. (Gly20Arg)* [=p. G(−2)R]	c.58 G > A	exon 1	*Fau I*	France, Austria, The Netherlands, New Zealand, Turkey, Croatia	1 (1)
*p. (Ser206Arg)* [=p.S185R]	c.618 T > G	exon 4B	*Bse21 I*	None (New)	1 (1)
*p. (Ser447Cys)* [=p. S426C]	c.1340C > G	exon 9	*BslFI*	None (New)	1 (2)
*p. (Leu511Ser)* [=p. L490S]	c.1532 T > C	exon 10B	*AclI*	Italy	1 (1)
*p. (Leu646Ile)* [=p. L625I]	c.1936C > A	exon 13	SSCP	None (New)	2 (2)
**Frameshift mutations**					
p. (Ser65Glyfs*64) [=FsS44:D108X]	c.192_201delinsGGACTTCA	exon 3	*Hph I*	None (New)	1 (2)
p. (Val66Cysfs*64) [=FsV45:D108X]	c.195_196insT	exon 3	SSCP	None (New)	1 (1)
p. (Pro309Lysfs*59) [=FsE287: V348X] (FH North Karelia)	c.925_931del	exon 6	*Bcc I*	Finland, Sweden, USA, St.Petersburg	1 (2)
p. (Trp562Cysfs*5) [=FsW541:L547X]	c.1686_1693delinsT	exon 11	*Hae III*	None (New)	1 (2)
p. (Val731Serfs*6) [=FsV710:V715X]	c.2191delG	exon 15	SSCP	None (New)	1 (2)
**Putative splice site mutations or neutral mutations**					
*p. (Asn591=)* [=p.N570N]	c. 1773C > T	exon 12	*Hinc II*	USA, China, Morocco, etc.	16%
**Neutral mutations/polymorphisms**					
*p. (Ala391Thr)* [=p. A370T]	c. 1171G > A	exon 8	*Stu I*	South Africa, England, France etc.	6%
**Silent mutations/polymorphisms**					
*p. (Ile398=)* [=p. I377I]	c.1194C > T	exon 9	*Bse3D I*	Austria	4 (4)
*p. (Arg471=)* [=p. R450R]	c.1413 G > A	exon 10A	*BslFI*	South Africa, Japan, Russia, Morocco etc.	34%
*p. (Pro539=)* [= p. P518P]	c.1617C > T	exon 11	*BspACI*	Morocco, China, Russia, etc.	5%
*p. (Asn640=)* [=p. N619N]	p.1920C > T	exon 13	*Sse 9 I*	Hispania, Austria.	1 (2)
*p. (Val653=)* [=p. V632V]	c.1959C > T	exon 13	*Asp S9 I*	The Netherlands, USA, Russia, etc.	49%
*p. (Arg744=)* [=p. R723R]	c.2232 G > A	exon 15	*Msp I*	Germany, China.	21%

Footnote: SSCP – single-strand conformation polymorphism analysis. Numeration of nucleotides and aminoacids follows modern nomenclature (numerals according to Yamamoto’s nomenclature [11] are given in brackets.

Footnote: Asterisk indicates that the codon with frameshift is followed by several non-wildtype codons up to newly appeared termination codon. For example, script p. (Trp562Cysfs*5) indicates that the frameshift occurs in codon 562 for tryptophan that is changed to codon for cysteine and is followed by 5 non-wildtype codons prior to termination codon.

## Discussion

Several novel mutant alleles that have been characterized in our study contain frameshifts, which results in occurrence of premature nonsense-codons (see Table 1). Besides detecting a well-characterized allele FH-North Karelia [12], a number of new mutations were identified in our study, including c.192_201delinsGGACTTCA (c.192del10/ins8), c.195_196insT, c.1686_1693del/insT (c.1686del8/insT) and c.2191delG. The deleterious character of these mutations seems likely since they cause a translation frameshift and formation of a premature termination codon (Table 1). Usual mechanism of action of a mutation introducing a premature codon is the nonsense-mediated decay of the corresponding transcript.

Of special interest, however, are missense-mutations that hamper the LDL receptor function. Analysis of these mutations (Additional file 3) by PROVEAN, PolyPhen, Mutation t@ster and SiftBlink gave somehow different predictions about pathogenicity of these missense mutations.

All of the methods agreed that *p. (Gly20Arg)* is a neutral variant. *p. (Gly20Arg)* mutation was first described among other disease-causing mutations by Amsellem et al. [13] in France and later reported from New Zealand [14], from the Netherlands [15], from Turkey [16] and from Austria [17]. Despite that *Gly-20* of the signal sequence is conserved in a number of animals, such as chimp, monkey, rat, rabbit and hamster, the most recent publications e.g. [18] consider it as a polymorphism. However, no segregation data were available and no direct functional analysis of the mutated protein was ever performed. In our sample *p. (Gly20Arg)* mutation was found in a genetic compound with very well characterized disease-causing mutation FH-North Karelia. Proband’s father carried only FH-North Karelia mutation and had quite typical atherogenic lipid plasma profile. In a reference patient *p. (Gly20Arg)* mutation was also present, but the profile was typical for heterozygous rather than homozygous FH. The mother of the genetic compound was not available for DNA analysis, but according to personal communication, she had no FH features. Therefore, these data are probably the first direct evidence that *p. (Gly20Arg)* mutation inherited by the proband from a healthy mother is a neutral non-FH causing variant.

Exact predictions regarding a function of the other missense mutations were not available using *in silico* analysis (Additional file 3). However, we believe that substitutions *p. (Ser447Cys)*, *p. (Leu646Ile)*, *p. (Ser206Arg)* and *p. (Leu511Ser)* resulted in the development of the FH phenotype. It’s important to note that *p. (Ser447Cys)* mutation introduces a novel cysteine residue, and it could affect specific Cys-Cys bonding in the EGF precursor homology domain of the LDL receptor. Another mutation, *p. (Ser206Arg)*, introduces a large positively charged side chain into strongly conserved ligand-binding repeat 5 (LR5) of the receptor. Mutation *p. (Leu646Ile)* doesn’t change the charge of the receptor molecule, but it provides for the substitution of the short aliphatic chain by the branched analogue that potentially could result in misfolding of the receptor protein. Allele *p. (Leu511Ser)* has previously been classified as a pathogenic variant FH Rome-4 in an Italian patient [19].

There were several variants in the LDL receptor exons predicted to be silent mutations due to preservation of the codon sense. However, a synonymous variant *p. (Asn591=)* (c. 1773C > T, also known as rs688) was shown not only to direct alternative splicing of mRNA followed by enhanced nonsense-mediated decay, but also to reduce the uptake of fluorescently labeled LDLs by 25%. This polymorphism was associated with increased plasma total and LDL cholesterol in several populations and so far can’t be considered a neutral nucleotide substitution [20]. However, *p. (Asn591=)* variant was found at a similar frequency in the St.Petersburg FH cohort and in the control group and thus unlikely is a disease-causing variant (M.Y. Mandelshtam, unpublished data).

No data suggesting functional role of other silent mutations have been published. This is also true for *p.(Ala391Thr)* (c.1171G > A variant), cited previously as p.A370T mutation or *Stu*I RFLP [21-23]. A minor allele of this polymorphism was found in a number of populations including Russian population [22], and it was demonstrated in major studies to have no influence on plasma lipid levels or cardiovascular risk in UK men [23]. Variant *p.(Pro539=)* (c. 1617C > T) was detected only once in St. Petersburg [7] among 80 FH probands and therefore, was regarded as a silent mutation. However, in the Petrozavodsk FH sample that rare allele occurs at a higher frequency (5%) and is further considered to be a DNA polymorphism. As shown in Table 1, *p.(Pro539=)* variation can be easily detected as *Aci*I (=*BspAC* I) RFLP. In case of minor allele the restriction site is lost; the PCR-amplified exon 11 of the LDLR gene is not cut and looks as a single band of 169 bp.

Other silent variants of the LDL receptor gene were previously characterized as neutral and were not associated with the disease; this is true for c.1413G > A or *p.(Arg471=)* polymorphism in exon 10A known as *BslFI* RFLP [24,25]), c.1959C > T transition or *p.(Val653=)* variant cited in many sources as *Ava*II RFLP or *Sau 96* I RFLP, and c.2232G > A or *p.(Arg744=)* transition known as *Msp*I RFLP [26]. All these variants have been reported both for Caucasian and Chinese populations [27].

## Conclusions

Considering epidemiology of FH in the Petrozavodsk population we can conclude that the FH cohort is very heterogeneous, showing no predominant FH-causing mutations. It seems that known mutations from Finnish population are only a rare cause of the disease in the Karelian FH sample; the same is true about the St. Petersburg FH collection [7]. Because no founder effect was detected in the Karelian FH sample, the rationale for FH diagnostics dictates direct sequencing of the full coding region of the gene and exon/intron boundaries rather than testing for known mutations. Modern DNA sequencing technologies allow performing such analysis quickly and at a relatively low price.

## Competing interests

The authors declare that they have no competing interests.

## Authors’ contributions

TYuKo, ASG, MYuM performed cloning, SSCP, sequencing and familial analysis, VAK and TYuKu selected patients for analysis, VBV and MYuM participated in the study design and coordination and also have written the paper. All authors read and approved the final manuscript.

## Pre-publication history

The pre-publication history for this paper can be accessed here:

http://www.biomedcentral.com/1471-2350/14/128/prepub

## Supplementary Material

Additional file 1 Lipid data of patients with LDL receptor mutations from Petrozavodsk sample. Click here for file

Additional file 2 Enzymes used in RFLP analysis for mutation validation. Click here for file

Additional file 3 In silico predictions of effects of the nucleotide substitutions found in the LDL receptor gene in Petrozavodsk FH sample. Click here for file
