# Supplementary material for: Familial hypercholesterolemia mutations in Petrozavodsk: no similarity to St. Petersburg mutation spectrum
Source: BMC Med Genet. 2013 Dec 27;14:128. doi: 10.1186/1471-2350-14-128 (PMC3877960; doi:10.1186/1471-2350-14-128)
Supplement: Additional file 1 — Lipid data of patients with LDL receptor mutations from Petrozavodsk sample. [file 1471-2350-14-128-S1.doc]

### Additional file 1 – Lipid data of patients with LDL receptor mutations from Petrozavodsk sample.

| Patient number | Sex | Age | Mutation (in the protein) | Xanthomata | Myocardial infarction | Total cholesterol, mM | Triglycerides, mM | LDL cholesterolmM | HDL cholesterol, mM | Ka* |
| --- | --- | --- | --- | --- | --- | --- | --- | --- | --- | --- |
| 10 | F | 56 | *p. (Leu511Ser)* | - | - | 16.1 | 2.7 | 14.1 | 0.7 | 22 |
| 19 | M | 37 | *p. (Ser447Cys)* | - | + | 9.9 | 2 | 7.6 | 0.6 | 15.5 |
|  |  |  | *p. (Leu646Ile)* |  |  |  |  |  |  |  |
| 20 | M | 11 | *p. (Asn640=)* | - | - | 5.6-6 | 2 | 4 | ND | ND |
| 23 | F | 4 | *p. (Ser447Cys)* | - | - | 7.8 | 1.1 | 5.9 | ND | ND |
|  |  |  | *p. (Asn640=)* |  |  |  |  |  |  |  |
| 26 | M | 28 | *p. (Val66Cysfs*64)* | - | - | 8.1 | 1.7 | 5 | 1.1 | 6.36 |
| 29 | M | 27 | *p. (Ser65Glyfs*64)* | - | - | 8 | 1.6 | 5.7 | 1.7 | 3.8 |
|  |  |  | *p. (Ile398=)* |  |  |  |  |  |  |  |
| 30 | F | 55 | *p. (Ser65Glyfs*64)* | xanthelasma | + | 10.7 | 1.2 | 8.2 | 1.5 | 6.1 |
| 38 | F | 26 | *p. (Trp562Cysfs*5)* | - | - | 8.8 | 1.4 | 5.95 | 2.0 | 3.4 |
| 39 | F | 29 | *p. (Trp562Cysfs*5)* | - | + | 8.4-9 | 0.8 | 6.4 | 1.3 | 5.7 |
|  |  |  | *p. (Ile398=)* |  |  |  |  |  |  |  |
| 42 | F | 55 | *p. (Val731Serfs*6)* | xanthelasma | - | 11.3-12 | 1.9 | 7.8 | 1.5 | 6.8 |
| 46 | F | 25 | *p. (Val731Serfs*6)* | - | - | 10.8 | 1.3 | 8.2 | 2 | 4.5 |
| 72 | F | 65 | *p. (Ser206Arg)* | xanthelasma | - | 13.7 | 1.2 | 11.4 | 1.7 | 6.9 |
| 74 | M | 55 | *p. (Pro309Lysfs*59)* | - | - | 11-14 | 3 | 7.7 | 1 | 11.4 |
| 75 | F | 21 | *p.* *(Gly20Arg)* | - | - | 7.8 | 0.9 | 6.3 | 1.1 | 6.1 |
|  |  |  | *p. (Pro309Lysfs*59)* |  |  |  |  |  |  |  |
| 88 | F | 69 | *p. (Ile398=)* | сorneal arcus, xanthelasma | + | 9.3 | 1.3 | 6.4 | 1.2 | 6.8 |
| 91 | M | 63 | p. *(Leu646Ile)* | - | - | 10.8 | 2.1 | 8.1 | ND | ND |
| 100 | F | 49 | *p. (Ile398=)* | - | - | 9.2 | ND | 6.7 | 2.1 | 3.4 |
