# Supplementary material for: Familial hypercholesterolemia mutations in Petrozavodsk: no similarity to St. Petersburg mutation spectrum
Source: BMC Med Genet. 2013 Dec 27;14:128. doi: 10.1186/1471-2350-14-128 (PMC3877960; doi:10.1186/1471-2350-14-128)
Supplement: Additional file 2 — Enzymes used in RFLP analysis for mutation validation. [file 1471-2350-14-128-S2.doc]

### Additional file 2 – Enzymes used in RFLP analysis for mutation validation.

| Prototype | Isoshizomer enzyme from Russian supplier |
| --- | --- |
| Fau I | *Fau I* |
| Hph I | *AsuHP I* |
| Sau96 I | *AspS9 I* |
| Sau I | *Bse21 I* |
| Bcc I | *BccI* |
| Stu I | *StuI* |
| Fin I | *BslF I* |
| BsrD I | *Bse3D I* |
| Acl I | *Acl I* |
| Aci I | *BspAC I* |
| Hae III | *Hae III* |
| Hinc II | *Hind II* |
| Tsp509 I | *Sse9 I* |
| Msp I | *Msp I* |
