# Supplementary material for: Familial hypercholesterolemia mutations in Petrozavodsk: no similarity to St. Petersburg mutation spectrum
Source: BMC Med Genet. 2013 Dec 27;14:128. doi: 10.1186/1471-2350-14-128 (PMC3877960; doi:10.1186/1471-2350-14-128)
Supplement: Additional file 3 — In silico predictions of effects of the nucleotide substitutions found in the LDL receptor gene in Petrozavodsk FH sample. [file 1471-2350-14-128-S3.doc]

**Additional file 3** - ***In silico* predictions of effects of the nucleotide substitutions found in the LDL receptor gene in Petrozavodsk FH sample.**

| **Mutation, systematic name**  **[name according to Yamamoto nomenclature] [26]** | **Nucleotide change (numeration according to reference sequence NM_000527)** | **PolyPhen2** | **Mutation T@ster** | **PROVEAN** | **SiftBlink** |
| --- | --- | --- | --- | --- | --- |
| *p.* *(Gly20Arg)*  [=p. G(-2)R] | c.58 G>A | benign | polymorphism | neutral | tolerated |
| *p.* *(Ser206Arg)*  [=p.S185R] | c.618T>G | benign | disease-causing | deleterious | not tolerated |
| *p.* *(Ser447Cys)*  [=p. S426C] | c.1340 C>G | probably damaging | disease-causing | deleterious | tolerated |
| *p.* *(Leu511Ser)*  [=p. L490S] | c.1532 T>C | probably damaging | disease-causing | deleterious | not tolerated |
| *p.* *(Leu646Ile)*  [=p. L625I] | c.1936 C>A | probably damaging | disease-causing | neutral | tolerated |
| *p. (Asn591=)*  [=p.N570N] | c. 1773 C>T | N/A | polymorphism | N/A | N/A |
| *p. (Ile398=)*  [=p. I377I] | c.1194C>T | N/A | disease-causing | N/A | N/A |
| *p. (Arg471=)*  [=p. R450R] | c.1413 G>A | N/A | polymorphism | N/A | N/A |
| *p. (Pro539=)*  [= p. P518P] | c.1617 C>T | N/A | polymorphism | N/A | N/A |
| *p. (Asn640=)*  [=p. N619N] | c.1920 C>T | N/A | polymorphism | N/A | N/A |
| *p. (Val653=)*  [=p. V632V] | c.1959 C>T | N/A | polymorphism | N/A | N/A |
| *p. (Arg744=)*  [=p. R723R] | c.2232 G>A | N/A | polymorphism | N/A | N/A |

Footnote: Numeration of nucleotides and aminoacids follows modern nomenclature (numerals according to Yamamoto’s nomenclature [26] are given in brackets. Programs to evaluate effects of mutations *in silico* were as follows: PolyPhen2 (genetics.bwh.harvard.edu/pph2); Mutation t@ster ([www.mutationtaster.org](http://www.mutationtaster.org/)); PROVEAN, v. 1.1.3 (provean.jcvi.org) and SiftBlink (sift.jcvi.org). N/A – not available.
